# Chitosan Alginate Nanoparticles of Protein Hydrolysate from *Acheta domesticus* with Enhanced Stability for Skin Delivery

**DOI:** 10.3390/pharmaceutics16060724

**Published:** 2024-05-28

**Authors:** Kankanit Yeerong, Panuwan Chantawannakul, Songyot Anuchapreeda, Saranya Juntrapirom, Watchara Kanjanakawinkul, Anette Müllertz, Thomas Rades, Wantida Chaiyana

**Affiliations:** 1 Department of Pharmaceutical Sciences, Faculty of Pharmacy, Chiang Mai University, Chiang Mai 50200, Thailand; kankanit_yeerong@cmu.ac.th; 2 Bee Protection Laboratory, Department of Biology, Faculty of Science, Chiang Mai University, Chiang Mai 50200, Thailand; panuwan.c@cmu.ac.th; 3 Division of Clinical Microscopy, Department of Medical Technology, Faculty of Associated Medical Sciences, Chiang Mai University, Chiang Mai 50200, Thailand; songyot.anuch@cmu.ac.th; 4 Center of Excellence in Pharmaceutical Nanotechnology, Faculty of Pharmacy, Chiang Mai University, Chiang Mai 50200, Thailand; 5 Chulabhorn Royal Pharmaceutical Manufacturing Facilities by Chulabhorn Royal Academy, Phlu Ta Luang, Sattahip, Chon Buri 20180, Thailand; saranya.jun@cra.ac.th (S.J.); watchara.kan@cra.ac.th (W.K.); 6 Department of Pharmacy, Faculty of Health and Medical Sciences, University of Copenhagen, Universitetsparken 2, 2100 Copenhagen, Denmark; anette.mullertz@sund.ku.dk (A.M.); thomas.rades@sund.ku.dk (T.R.); 7 Bioneer: FARMA, Department of Pharmacy, University of Copenhagen, Universitetsparken 4, 2100 Copenhagen, Denmark; 8 Multidisciplinary and Interdisciplinary School, Chiang Mai University, Chiang Mai 50200, Thailand

**Keywords:** *Acheta domesticus*, house cricket, protein hydrolysate, chitosan, alginate, nanoparticles, skin delivery, cosmetic, cosmeceutical, enhanced stability

## Abstract

This study aimed to develop chitosan alginate nanoparticles (CANPs) for enhanced stability for dermal delivery of protein hydrolysate from *Acheta domesticus* (PH). CANPs, developed using ionotropic pre-gelation followed by the polyelectrolyte complex technique, were characterized for particle size, polydispersity index (PDI), and zeta potential. After the incorporation of PH into CANPs, a comprehensive assessment included encapsulation efficiency, loading capacity, morphology, chemical analyses, physical and chemical stability, irritation potential, release profile, skin permeation, and skin retention. The most optimal CANPs, comprising 0.6 mg/mL sodium alginate, 1.8 mg/mL calcium chloride, and 0.1 mg/mL chitosan, exhibited the smallest particle size (309 ± 0 nm), the narrowest PDI (0.39 ± 0.01), and pronounced negative zeta potential (−26.0 ± 0.9 mV), along with an encapsulation efficiency of 56 ± 2%, loading capacity of 2.4 ± 0.1%, release of 40 ± 2% after 48 h, and the highest skin retention of 12 ± 1%. The CANPs induced no irritation and effectively enhanced the stability of PH from 44 ± 5% of PH remaining in a solution to 74 ± 4% after three-month storage. Therefore, the findings revealed the considerable potential of CANPs in improving PH stability and skin delivery, with promising applications in cosmetics and related fields.

## 1. Introduction

Over the past few years, there has been a notable surge of interest surrounding bioactive peptides in the cosmetic industry, driven by their potential to improve skin health and enhance beauty [1]. These peptides display diverse biological activities, encompassing antioxidant [2], anti-skin aging [3], anti-inflammatory [4], and antimicrobial properties [5], rendering them promising for integration into cosmetic formulations. Many researchers have actively sought new sources of these peptides, aiming for cost-effectiveness and sustainability [6]. Among these novel sources, *Acheta domesticus*, commonly known as the house cricket, has emerged as an appealing protein source for bioactive peptides specifically targeting anti-aging skin benefits [7]. Our previous study discovered that the protein hydrolysate obtained from *A. domesticus* (PH), prepared with Alcalase^®^, exhibited noteworthy activities against skin aging, including collagenase inhibition, hyaluronidase inhibition, radical scavenging, lipid peroxidation inhibition, and anti-NF-κB-mediated inflammation [8]. Despite its potent bioactivities, PH faces limitations as an active ingredient in cosmetic/cosmeceutical products due to its instability, e.g., the Maillard reaction during thermal processing can affect both the technological-functional characteristics and bioactive properties through protein cross-linking, glycation, and aggregation, thereby promoting proteolytic degradation [9]. Additionally, pH and salt significantly impact the physicochemical properties and structure of *A. domesticus* protein, affecting solubility, water-holding, emulsion-forming, and foaming capacities [10,11]. Regarding the potential proteolytic degradation from diverse factors, novel strategies to protect the PH against adverse degradation would be essential to ensure stability and sustained effectiveness.

Polymeric nanoparticles (PNPs) have been extensively used as novel drug delivery systems for proteins and peptides due to their promising protection from environmental stresses [12], such as temperature, pH, oxidation, heating, and ultraviolet light [13,14]. Additionally, PNPs could improve the bioavailability and efficacy of encapsulated compounds due to their small size, large surface area, potential targeted delivery, and controlled release properties [12]. Various polymers, both synthetic and natural, can be utilized for preparing PNPs [15]. However, synthetic polymers like poly(ε-caprolactone), poly(lactic acid), and their copolymers are suboptimal carriers for hydrophilic peptides and proteins due to their hydrophobic properties [16]. Additionally, the use of organic solvents in the preparation process may diminish the biological activities of proteins. In contrast, natural polymers play a crucial role in crafting desirable nanoscale carriers for proteins or peptides, given their hydrophilicity, biodegradability, biocompatibility, and the avoidance of harsh conditions in the preparation process [17]. Therefore, natural biodegradable polymers, such as alginate and chitosan, are attractive in cosmetics applications that are generally non-reactive when in contact with the human skin and can be broken down or metabolized and removed from the body via normal metabolic pathways [18]. 

Chitosan alginate nanoparticles (CANPs) serve as a drug delivery system employing natural polymers, including sodium alginate and chitosan. Alginate is a linear anionic polysaccharide derived from brown algae, comprised varying proportions of 1,4-linked β-D-mannuronic acid (M) and α-L-glucuronic acid (G) [19]. Sodium alginate is a sodium salt form of alginic acid, which is widely used in cosmetic formulation due to its gel formation ability, biocompatibility, non-toxicity, biodegradability, and mucoadhesion [20,21,22]. In addition, sodium alginate has been employed in nano-delivery applications, forming gels with divalent metal ions such as calcium ions (Ca^2+^) under mild conditions, making it an excellent candidate for delivering proteins or peptides and minimizing the risk of denaturation [23,24]. However, the instability and loss of encapsulated active compounds in sodium alginate PNPs can be solved by adding aqueous polycationic solutions like chitosan to form a polyelectrolyte complex, thereby enhancing PNP structural strength [25,26]. Chitosan is a natural cationic linear polysaccharide derived from chitin via a deacetylation reaction in an alkaline solution and composed of randomly distributed β-(1 → 4)-linked d-glucosamine and *N*-acetyl-d-glucosamine [27]. Chitosan has been used in topical and transdermal delivery systems due to its non-toxicity, biocompatibility, and biodegradability [28], as well as its various bioactivities beneficial to the skin, including antioxidant [29], antibacterial [30], antifungal [31], anti-proinflammatory cytokines [32], and accelerating dermal regeneration [33]. Furthermore, chitosan could enhance permeation across the skin by interacting with negatively charged cells in the stratum corneum layer, which may alter the secondary structure of keratin and widen the tight junctions to create a looser skin structure, ultimately leading to enhanced transdermal permeation of active compounds [34,35]. The combination of chitosan and sodium alginate is imperative to fortifying the structure of PNPs, establishing a dense structure that effectively regulates rapid diffusion, particularly in acidic pH ranges, promotes sustained release, and has the potential to shield entrapped active molecules from oxidation, enzymatic degradation, and hydrolysis [36,37,38]. CANPs have diverse applications in protein and peptide delivery, such as insulin [39], measles antigens [40], bovine serum albumin (BSA) [41], etc. CANPs exhibited various advantages, including an optimal particle size range, outstanding encapsulation capacity and loading capacity, safety in both in vitro and in vivo systems, protective, sustained-release kinetics, and enhanced efficacy [39,40,41].

In our previous study, the process for extracting PH from *A. domesticus* has been optimized and its potential cosmeceutical effects have been reported [8]. Therefore, the objectives of the present study were to develop CANPs aimed at enhancing the stability of PH derived from *A. domesticus* and investigating their effectiveness in improving skin delivery for potential applications in cosmeceuticals/cosmetic products.

## 2. Materials and Methods

### 2.1. Chemical Reagents

Low molecular weight chitosan (MW = 50,000–190,000 Da with 75–85% degree of deacetylation), low-viscosity sodium alginate from brown algae (MW = 30,000–100,000 Da), BSA, sodium phosphate monobasic monohydrate (NaH_2_PO_4_•H_2_O), potassium bromide (KBr, FT-IR grade), cupric sulfate, and sodium dodecyl sulfate (SLS) were purchased from Sigma Aldrich in St. Louis, MO, USA. Glacial acetic acid, hydrochloric acid (HCl), sodium hydroxide (NaOH), sodium chloride (NaCl), and calcium chloride (CaCl_2_) were analytical grade and obtained from RCI Labscan Ltd. in Bangkok, Thailand. Disodium hydrogen phosphate (Na_2_HPO_4_) was purchased from Fisher Chemicals in Loughborough, UK. Bicinchoninic acid (BCA) protein assay kit and Alcalase^®^ enzyme from *Bacillius licheniformis* were purchased from Merck Ltd. (Darmstadt, Germany).

### 2.2. Fabrication of CANPs

CANPs were prepared using an ionotropic pre-gelation technique with calcium chloride followed by a polyelectrolyte complex method, as reported by Azevedo et al., with some modifications [42]. Briefly, sodium alginate was dissolved in distilled water, and chitosan was dissolved in 1% *v*/*v* acetic acid, with pH values of the solutions adjusted to 4.9 and 4.6, respectively. Additionally, the calcium chloride solution was freshly prepared by dissolving it with distilled water. All solutions underwent filtration through 0.22 μm cellulose acetate membrane filters (Sartorius Stedim Biotech GmbH, Göttingen, German) for insoluble impurity removal. In the first step, the calcium chloride solution was dropped into sodium alginate solution through 31G needles (Nipro, Osaka, Japan) while being stirred at 600 rpm for 90 min with a multi-position magnetic stirrer (EOS Scientific Co., Ltd., Bangkok, Thailand), resulting in the pre-gelation solution. Subsequently, the pre-gelation solution was sonicated using an ultrasonic bath (Elma, Fremont, CA, USA) for 15 min. The chitosan solution was added dropwise into the calcium alginate pre-gel solution under continuous stirring for an additional 90 min and then sonicated for 15 min to enhance uniformity of the dispersed particles. After sonication, the resulting mixture was homogenized using a high-shear homogenizer (Ultra-Taurrax T25 Basic IKA-Works Inc., Wilmington, NC, USA) at 10,000 rpm for 15 min and allowed to equilibrate at 4 °C overnight. After incubation, any excess polymer was eliminated from CANPs using ultrafiltration membranes with a 10 kDa molecular weight cutoff (Amicon^®^, Sigma Aldrich, St. Louis, MO, USA) through centrifugation using a laboratory centrifuge (MPW-352R, MPW Med. Instruments, Warsaw, Poland). The retentate was collected, resulting in the final CANPs formulation.

### 2.3. Optimization of Process Parameters for CANPs Production

To achieve CANPs with desirable characteristics, such as a small particle size, narrow size distribution, and proper zeta potential, the influences of process parameters, such as sodium alginate, calcium chloride, and chitosan concentrations were investigated through the one-factor-at-time method [43]. The concentrations of sodium alginate (0.1, 0.2, 0.4, 0.6, 0.8, and 1.0 mg/mL), the concentration of calcium chloride solution (1.3, 1.6, 1.8, 2.0, 2.2, and 2.4 mg/mL), and the concentration of chitosan (0.1, 0.2, 0.4, 0.6, 0.8, and 1.0 mg/mL) were examined for their effects on CANPs characteristics, including particle size, polydispersity index (PDI), and zeta potential. While varying one parameter, the others were held constant. Subsequently, suitable variables were selected to develop CANPs-incorporated *A. domesticus* PH.

### 2.4. Preparation of PH Using Enzymatic Hydrolysis

PH was prepared by enzymatic hydrolysis following our previous method [8]. In brief, *A. domesticus* were extracted using distilled water at 45 °C for 3 h using a water bath (Memmert, Schwabach, Germany), and then proteins were precipitated by isoelectric precipitation. After that, the proteins were redispersed in distilled water, followed by adjustment to pH 7.5 using 1 M NaOH solution. The enzymatic hydrolysis was carried out using Alcalase^®^ with the optimized condition (E/S concentration of 2.1% (*w*/*w*), 227 min, and 61.5 °C). To stop the hydrolysis reaction, the enzyme was deactivated by heating at 90 °C for 20 min in a water bath and then subjected to centrifugation at 8000× *g* for 15 min. The resulting supernatant was collected and dried using a freeze dryer (Beta 2–8 LD-plus, Martin Christ Gefriertrocknungsanlagen GmbH, Osterode am Harz, Germany), yielding PH, which was stored at −20 °C for further analysis.

### 2.5. Fabrication of PH-Loaded CANPs

The CANPs formulations exhibiting favorable characteristics, including a small particle size, narrow PDI, and appropriate zeta potential, were selected for the incorporation of PH following the study of Zohri et al. [41]. From our previous study, the concentration at half-maximal (IC_50_) biological activities against skin aging of PH was observed at 122 μg/mL [8]. Therefore, a concentration of 10 times the IC_50_ value (12.5 mg/mL) was selected for incorporation into CANPs. Briefly, PH was blended into the sodium alginate solution before the addition of the calcium chloride solution under continuous stirring at 600 rpm for 30 min. Following this step, the PH-loaded CANPs were prepared using the same CANPs preparation method previously described. Subsequently, the PH-loaded CANPs were also characterized, with respect to particle size, PDI, and zeta potential.

### 2.6. Determination of Total Protein Content of PH

PH was investigated for total protein content using a BCA assay according to Yeerong et al.’s study [7]. In essence, the BCA working solution was freshly prepared by combining 200 μL of BCA reagent with 4 mL of 4% cupric sulfate solution. Subsequently, 25 μL of the sample was mixed with 200 μL of BCA working solution and incubated at 37 °C for 30 min. The resulting mixture was measured for absorbance at 562 nm using a multimode detector (BMG Labtech, Ortenberg, Germany). A calibration curve was established using BSA concentrations ranging from 0.1 to 1.0 mg/mL. The protein content of each sample was determined by comparing it to the BSA standard curve. All experiments were independently conducted in triplicate.

### 2.7. Determination of Encapsulation Efficiency and Loading Capacity of PH-Loaded CANPs

Encapsulation efficiency and loading capacity of PH-loaded CANPs were determined following Sorasitthiyanukarn et al.’s study (2021) with some modifications [44]. Briefly, each PH-loaded CANPs formulation was subjected to the Amicon^®^ Ultra-15 centrifugal filter device (Merck, Darmstadt, Germany) and centrifuged at 5000× *g* for 15 min. Following centrifugation, a filtrate containing free PH was collected and quantified for total protein content using a BCA assay as described above. The percentage of encapsulation efficiency of the PH-loaded CANPs was calculated using the following equation:

Encapsulation efficiency (%) = 100 × (1 − B/A),(1)

where A represents the total protein amount of PH and B represents the free protein amount in filtrate. In addition, the percentage of loading capacity of the PH-loaded CANPs was calculated using the following equation:

Loading capacity (%) = 100/C × (A − B),(2)

where A represents total protein amount of PH, B represents free protein amount in filtrate, and C represents total dry mass of PH-loaded CANPs obtained after freeze drying. All experiments were conducted in triplicate, and the results were expressed as mean ± standard deviation (SD). 

### 2.8. Stability Study of CANPs and PH-Loaded CANPs

CANPs and PH-loaded CANPs were assessed for accelerated stability through 8 cycles of heating–cooling conditions and long-term storage at various temperatures (4 °C, 25 °C, and 45 °C) over a period of 90 days [45]. Each cycle of heating–cooling was composed of 4 °C for 24 h and then 45 °C for 24 h. Physical and chemical stability were examined after heating–cooling conditions and during long-term storage at 30, 60, and 90 days. The physical stability was assessed in terms of physical appearance, particle size, PDI, and zeta potential. The chemical stability was determined for total protein content using a BCA assay as described above. Each investigation, except for the assessment of physical appearance, was conducted in triplicate, and the results are presented as the mean ± SD.

### 2.9. Physicochemical Characterization of CANPs

#### 2.9.1. Particle Size, Particle Size Distribution, and Zeta Potential Analysis 

CANPs and PH-loaded CANPs were investigated for particle sizes, particle size distributions, and zeta potentials using the dynamic light scattering (DLS) method with a Zetasizer (Nano-ZS90 model, Malvern Instruments, Malvern, UK). Before analysis, each sample was diluted with distilled water at a ratio of 1:10. The analysis was conducted at a scattering angle of 90°, a refractive index of 1.590, and a temperature of 25 °C. The particle size was reported as z-average, the particle size distribution was expressed as PDI, and the surface charge of the particles was reported as zeta potential.

#### 2.9.2. External Appearance and Morphological Analysis 

The external characteristics of CANPs and PH-loaded CANPs formulations were examined using organoleptic analysis [46]. The morphologies of CANPs and PH-loaded CANPs were visualized using Transmission Electron Microscopy (TEM) (Hitachi, HT7700, Hitachi, Ibaraki, Japan) with a negative staining technique according to the study of Lertsutthiwong et al. (2009) [47]. Briefly, each sample was diluted with distilled water in the ratio of 1:20 before the experiment. A total of 1.4 μL of the mixture was applied directly to a carbon film (300 mesh Cu/Ni grid filmed with carbon, Ted Pella Inc., Redding, CA, USA) and incubated for 3 min. The excess solution was wicked out with filter papers (Sartorius AG, Göttingen, Germany). The grid was then negatively stained with 1% uranyl acetate (EMS, Hatfield, PA, USA), which was dripped onto the grid surface tilted at 45° for 3 min. Viewing occurred at magnifications ranging from 25,000 to 50,000×, and the TEM operation was set at 120 kV.

#### 2.9.3. Fourier Transform Infrared (FT-IR) Spectroscopy 

The different types of CANPs and PH-loaded CANPs were investigated for chemical bonds and potential interactions among the polymers and PH using FT-IR spectroscopy equipped with the single reflection diamond ATR module (Bruker, ALPHA II, Bremen, Germany) [48]. For this analysis, the pure polymers (chitosan or sodium alginate), PH, freeze-dried CANPs, freeze-dried PH-loaded CANPs, and its physical mixture were placed on the diamond crystal, and the pressure tip was then lowered to press the sample powder closely against the diamond crystal. This ensured that the IR beam could pass through the sample above the crystal’s top surface. Subsequently, the IR spectrum was scanned and recorded within the wavenumber range of 400–4000 cm^−1^. The resulting FT-IR spectra were graphed with transmittance on the Y-axis and wavenumber (cm^−1^) on the X-axis.

#### 2.9.4. Differential Scanning Calorimetry (DSC)

CANPs and PH-loaded CANPs were characterized for thermal properties and elucidated interactions between polymers and PH using DSC following the method from Ribeiro et al. (2005) [49]. Chitosan, sodium alginate, PH, freeze-dried CANPs, freeze-dried PH-loaded CANPs, and its physical mixture were investigated during heating from 25 to 300 °C with a heating rate of 10 °C per min under an inert nitrogen atmosphere with a DSC1 star system (Mettler Toledo, Greifensee, Switzerland).

### 2.10. Irritation Test by Hen’s Egg Test on the Chorioallantoic Membrane (HET-CAM) Assay 

CANPs and PH-loaded CANPs were investigated for potential irritation using the HET-CAM test, following the method described by Tammasorn et al. (2023) [50]. Due to the intention of using these formulations in cosmetics, it is essential to assess their irritative properties before application. HET-CAM is recognized as a method for assessing irritation of test chemicals, particularly relevant for formulations intended for use in delicate areas, by observing adverse changes in the CAM of fertilized hen’s eggs (Steiling et al., 1999; Somwongin et al., 2018). The irritation signs, including hemorrhage, vascular lysis, and coagulation, were monitored under a stereo microscope (Olympus, Tokyo, Japan) over 60 min. For each irritation sign, the onset was noted, and the irritation score (IS) was calculated using the following equation:

IS = [(301 − h) × 5]/ 300 + [(301 − l) × 7]/300 + [(301 − c) × 9]/300,(3)

where h is timespan (s) of the first hemorrhage, l is timespan (s) of the first vascular lysis, and c is timespan (s) of the first coagulation. The IS were categorized into four groups: non-irritation (0.0–0.9), modest irritation (1.0–4.9), moderate irritation (5.0–8.9), and severe irritation (9.0–21.0). The experiment was conducted in duplicate, and the results are presented as the mean ± SD. SLS aqueous solution (1% *w*/*v*) and normal saline solution (0.9% (*w*/*w*) NaCl) were used as positive and negative controls, respectively.

### 2.11. In Vitro Release Study of PH-Loaded CANPs

The release study of PH from PH-loaded CANPs was conducted according to the method outlined by Jiamphun and Chaiyana (2023) with some modifications [45]. In brief, 1 mL of PH-loaded CANPs was introduced into a dialysis bag (Spectra/Por 1 Dialysis Tubing, nominal molecular weight cut off 6000–8000 Da, Fisher Chemicals, Loughborough, UK). After that, the dialysis bag was immersed in 25 mL of phosphate buffered saline (PBS) solution with a pH of 5.5. The temperature was maintained at 32 °C under continuous stirring at 250 rpm with a multi-position magnetic stirrer (AM4, EOS Scientific Co., Ltd., Bangkok, Thailand). Sampling was conducted at various time points (0.5, 1, 2, 3, 6, 8, 12, 24, and 48 h), and upon each sampling, the withdrawn sample was promptly replaced with an equivalent volume of fresh PBS solution. The collected samples were then analyzed for total protein content using the BCA assay, as previously described. PH solution was used as a control. The protein contents of released PH from PH-loaded CANPs were investigated and compared with the result from the PH solution. All formulations were investigated in duplicate, and the results were presented as the mean ± SD.

### 2.12. Skin Permeation and Skin Retention Study of PH-Loaded CANPs

#### 2.12.1. Skin Permeation Determination

The skin permeation of PH from PH-loaded CANPs was investigated using Franz diffusion cells (Velp Scientific Inc., Milano, Italy), following the method by Neimkhum et al. (2023) [51]. In brief, 12 mL of receptor media (PBS with a pH of 7.4) was thermostated at 37 °C and stirred consistently at 200 rpm using a Franz diffusion cell apparatus (Velp Scientific Inc., Milano, Italy). To avoid interference of protein content from the skin, synthetic artificial membranes for transdermal diffusion testing (Strat-M^®^, Merck Millipore, Burlington, MA, USA) were selected for the experiment. These membranes are designed to mimic human skin, with the outer layer consisting of two layers of polyethersulfone (PES), providing greater hydrophobicity, while the bottom layer is more hydrophilic [52]. The membranes were mounted on the apparatus for 30 min before the experiment to ensure equilibration. Subsequently, 200 μL of each sample was placed in the donor compartment. Aliquots of 1 mL of each receptor medium were withdrawn at various time points (0.5, 1, 2, 3, 6, 8, 12, 24, and 48 h). Once the PBS medium was withdrawn, it was immediately replaced with an equal amount of new PBS medium. An aqueous solution of PH was used as a control. The protein contents of permeated PH from PH-loaded CANPs through the skin were determined by the BCA assay and were compared with the result from the PH solution. Each experiment was conducted in duplicate, and the results are presented as the mean ± SD.

#### 2.12.2. Skin Retention Determination

CANPs and PH-loaded CANPS were investigated for skin retention using a method previously described by Marsup et al. (2020) with some modifications [53]. After 48 h of sample application, the membranes from the skin permeation study were collected to assess the retained total protein content from PH-loaded CANPs and PH solution. Consequently, the membranes were washed three times with PBS and then cut into small pieces. One milliliter of PBS was added, and the mixture was subjected to sonication to extract the proteins from the membranes. The protein contents of retained PH from PH-loaded CANPs were determined using the BCA assay and were compared with the result from the PH solution. Each investigation was conducted in duplicate, and the results are presented as the mean ± SD.

### 2.13. Statistical Analysis

The data are presented as the mean ± SD and statistical analyses were conducted using GraphPad Prism (version 8.0, GraphPad Software, San Diego, CA, USA). Paired sample *t*-tests and One-way Analysis of Variance (ANOVA) were performed to determine statistical significance. The level of statistical significance was set at *p* < 0.05.

## 3. Results and Discussions

### 3.1. Fabrication of CANPs

The influence of several factors on CANPs has been evaluated, including the effects of sodium alginate, calcium chloride, and chitosan, as shown in Table 1. The particle size of CANPs increased gradually with higher sodium alginate concentrations, while the PDI decreased until reaching 0.4 mg/mL of sodium alginate, after which the PDI remained constant. Additionally, it was found that the zeta potential of CANPs shows an increment in a negative way with increasing sodium alginate concentration, due to the negative charge of sodium alginate. At a pH above pKa of sodium alginate (~3.5), carboxyl (COOH) groups from sodium alginate monomers underwent ionization [54]. This ionization facilitated ionic interactions with Ca^2+^ from calcium chloride, resulting in pre-gelation and nucleus formation of CANPs. As the concentration of sodium alginate increased, there was a corresponding rise in ionic interactions with Ca^2+^, leading to the thickening of the alginate coating around the nucleus and an increase in the particle size of CANPs. Furthermore, the escalating sodium alginate concentration led to an imbalance between the negatively charged groups from sodium alginate and the positively charged groups from calcium chloride and chitosan, contributing to the negative zeta potential of CANPs [43]. The negative zeta potential introduced repelling forces that hindered particle aggregation, thereby ensuring the stability of the system [38]. Nevertheless, with a progressive decrease in charge, polymer chains underwent dissociation, resulting in the formation of larger and less dense particles [38]. The obtained results aligned well with previous research, which revealed that the ratio of sodium alginate, especially for G content, served as the primary factor controlling the physical properties of CANPs [55]. Therefore, sodium alginate concentration at 0.6 mg/mL was selected for further CANPs development due to its favorable outcomes, including appropriate particle size (288 ± 5 nm), the smallest PDI value (0.40 ± 0.01), and the most negative zeta potential value (−27.8 ± 2.3). 

Besides the sodium alginate, calcium chloride, which was used as a cross-linker, also affected the CANPs. In contrast to sodium alginate, where higher concentrations resulted in larger CANP particle sizes, the optimum concentration of calcium chloride was observed at 1.8 mg/mL. Below 1.8 mg/mL calcium chloride concentrations, CANPs particle size decreased with rising calcium chloride concentration and showed a slight increase at concentrations beyond 2.0 mg/mL. This trend was consistent with the observed PDI and zeta potentials. Remarkably, the CANPs prepared with a calcium chloride concentration of 1.8 mg/mL exhibited the significantly smallest particle size and the narrowest PDI, along with the lowest zeta potential (*p* < 0.05). This phenomenon could be attributed to the optimal bonding of Ca^2+^ to COO^−^ of sodium alginate, combined with chelation with the protonated amino (NH_3_^+^) of chitosan [56]. In general, calcium chloride played a pivotal role in CANPs formation by providing a nucleus for CANPs growth with sodium alginate in a pre-gel state [37]. The interaction between Ca^2+^ and negatively charged residues of sodium alginate resulted in the formation of compact egg-box structures in the nucleus [47]. The subsequent addition of chitosan stabilized the system by enveloping the particles, resulting in the formation of solid and small CANPs [57]. Nevertheless, an excess of Ca^2+^ induced intermolecular crosslinking, causing more ionotropic gel formation and the system to become more viscous, leading to an increase in particle size and aggregation [55]. This finding is supported by Sinjan and Robinson’s study, which observed that an excess of Ca^2+^ could induce rapid gelation through increased ionic interaction leading to larger particle sizes [37]. In contrast to the significant impact of calcium chloride addition on the particle size and PDI of CANPs, only minor effects were observed on the zeta potential values, which ranged from −24.3 ± 0.6 to −27.8 ± 0.1 mV. Although zeta potential generally decreases with increasing ionic strength due to the compression of the electrical double layer, slight effects were observed in the present study due to the low concentration of calcium chloride used in the formulations (1.3 to 2.4 mg/mL, accounting for only 0.012 to 0.022 M). As a result, a calcium chloride concentration of 1.8 mg/mL was selected for further CANPs development, demonstrating the significantly smallest particle size, a narrow PDI, and the lowest zeta potential value (*p* < 0.05) with values of 269 ± 5 nm, 0.29 ± 0.00, and −27.8 ± 0.1 mV, respectively.

On the other hand, chitosan was found to affect CANPs in a similar manner to sodium alginate. The particle size and PDI of CANPs showed a gradual increase with rising chitosan concentration, and the significantly smallest particle size and the narrowest PDI were observed at chitosan concentrations of 0.1, 0.2, and 0.4 mg/mL (*p* < 0.05). At higher chitosan concentrations, the particle sizes became larger, and the PDI also exhibited higher values. This increase in particle size might be attributed to the formation of multiple chitosan coating layers through electrostatic complexation with the sodium alginate nucleus [58]. Furthermore, it was observed that the zeta potential of CANPs increased with higher chitosan concentrations. This phenomenon may be attributed to the characteristic positive surface charge of chitosan. At the pH of CAPs preparation, the amino groups of chitosan (pKa = 6.5) are protonated, acquiring a positive charge [59]. These positively charged groups then neutralized the negative charge of the sodium alginate, leading to an overall increased zeta potential of the CANPs. This result was consistent with a previous study, which found that an increased chitosan/sodium alginate ratio in the CANPs preparation (from 1:4 to 4:1) led to larger particle sizes [58]. Moreover, the excess amount of polymer also contributed to the formation of particle clusters, resulting in aggregation and an unstable system [60]. 

In summary, the optimal formulations selected for the development of CANPs incorporating PH were prepared with a sodium alginate concentration of 0.6 mg/mL, a calcium chloride concentration of 1.8 mg/mL, and chitosan concentrations of 0.1, 0.2, and 0.4 mg/mL, respectively.

### 3.2. Fabrication of PH-Loaded CANPs

#### 3.2.1. Effect of Incorporating PH on CANPs Characteristics

PH was found to have a significant impact on CANP characteristics, particularly in terms of particle size (*p* < 0.05), as exhibited in Table 2. All PH-loaded CANP formulations (formulation numbers 4 (F4), 5 (F5), and 6 (F6)) showed marginally larger particle sizes than the respective blank CANP formulations (formulation numbers 1 (F1), 2 (F2), and 3 (F3)). The significantly smallest particle size was observed in F4 (*p* < 0.05), which was prepared using the lowest chitosan concentration (0.1 mg/mL), whereas the PDI remained constant across the various other formulations. Furthermore, the zeta potential of F4, F5, and F6 slightly decreased upon the incorporation of PH compared to F1, F2, and F3. Our previous study revealed that PH is rich in various amino acids, particularly negatively charged amino acids such as glutamic acid and aspartic acid [8]. Therefore, it could be assumed that the interaction between PH and CANPs is primarily ionic. The COO^−^ group in the amino acid sequences of PH could interact with positively charged Ca^2+^. Subsequently, the positively charged chitosan formed a complex with free negatively charged sodium alginate and PH through ionic bonds, contributing to the stabilization of the CANPs structure [60]. Additionally, hydrogen bonding and Van der Waals forces were potentially involved in the interaction between PH and the polymers [61].

This finding aligned with a study by Sorasitthiyanukarn et al. (2021), who used a similar CANPs formulation to incorporate curcumin diethyl disuccinate (CDD) [44]. The study employed a sodium alginate concentration of 0.6 mg/mL, a calcium chloride concentration of 0.67 mg/mL, and a chitosan concentration of 0.15 mg/mL to incorporate 1.5 mg/mL of CDD, with the sodium alginate/calcium chloride/chitosan mass ratio fixed at 1:0.22:0.05. The reported physical characteristics of CDD-loaded CANPs in that study were a particle size of 340 ± 14 nm, a PDI of 0.37 ± 0.02, and a zeta potential of −28.2 ± 0.8 mV. However, Loquercio et al. (2015) found the smaller CANPs entrapped trans-cinnamaldehyde, with a size of around 166 nm and a PDI of 0.57, which were prepared using 0.06% *w*/*v* sodium alginate, 0.2% *w*/*v* calcium chloride, and 0.05% *w*/*v* chitosan, with a fixed mass ratio of 1:0.21:0.67 [38]. The physical characteristics of the developed CANPs were found to vary, depending on many factors such as polymer characteristics, pH of the preparation, final concentration of polymer solutions, mass ratio of polymer, cross-linker concentration, preparation techniques, characteristics of the encapsulated substances, and size reduction methods [38,55]. Consequently, optimizing the CANPs preparation method was deemed crucial for designing effective nanoparticle systems to encapsulate each unique active compound.

#### 3.2.2. Encapsulation Efficiency and Loading Capacity of PH-Loaded CANPs

**Figure 1:**
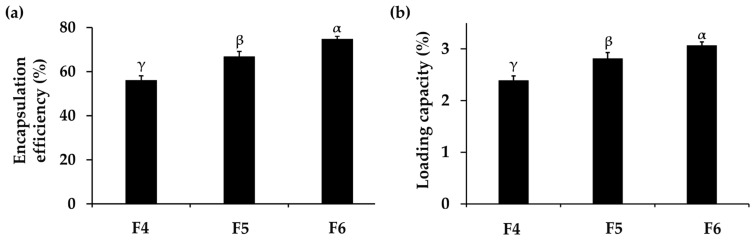
Encapsulation efficiency (**a**) and loading capacity (**b**) of *A. domesticus* protein hydrolysate (PH) from PH-loaded chitosan alginate nanoparticles (CANPs), including formulations F4, F5, and F6, respectively. Different letters, α, β, and γ, denoted significant differences in encapsulation efficiency and loading capacity among the various PH-loaded CANPs formulations analyzed using a One-way ANOVA followed by a post hoc Tukey test (*p* < 0.05).

The encapsulation efficiency and loading capacity of F4, F5, and F6 were investigated, and the results are shown in Figure 1. These parameters are crucial as they implied the amount of drug entrapped in the nanoparticles, thereby influencing stability, absorption, bioavailability, and therapeutic efficacy [62]. The results showed that the encapsulation efficiency of PH-loaded CANPs formulations ranged from 56 ± 2% to 75 ± 1% and the loading capacity of PH-loaded CANPs ranged from 2.4 ± 0.1% to 3.1 ± 0.1%, respectively. The significantly highest encapsulation efficiency and loading capacity were observed in F6 (*p* < 0.05), followed by F5 and F4, respectively. It was noted that the encapsulation efficiency and the loading capacity increased with the increase in chitosan concentrations. The reason might be due to more self-assembly formation through ionic interactions between the positively charged chitosan and the negatively charged PH present in the alginate–calcium intermolecular complexes, consequently enhancing the encapsulation efficiency and loading capacity [39]. Moreover, the elevated chitosan concentration could act as a shield by forming coating layers on the alginate–calcium complexes, preventing the leakage of the entrapped drug, and resulting in encapsulation improvement [63]. Although the encapsulation efficiency in this study was not very high, it could be attributed to the competitive interaction between sodium alginate and PH [64]. When PH was mixed with the sodium alginate solution under acidic conditions, both sodium alginate and PH were negatively charged. They competitively interacted with positively charged Ca^2+^ and chitosan, hindering the encapsulation of PH into the CANPs [65]. The results in the present study are consistent with several previous studies on CANPs incorporating various types of proteins. Maryam et al. (2011) also reported that the encapsulation efficiency of BSA-loaded CANPs ranged from 60% to 70% by varying the chitosan to sodium alginate ratios [41]. Additionally, a study conducted by Mukhopadhyay et al. (2015) also observed that the encapsulation efficiency of insulin in CANPs increased as the weight ratios of chitosan were elevated [39]. Furthermore, a previous study demonstrated loading capacities of sodium alginate-coated with low molecular weight chitosan nanoparticles incorporating measles antigens, ranging from 1.2 to 3.4% [41], with values corresponding to the findings in this study. In addition, Sarmento et al. (2007) revealed that the association efficiency and loading capacity of insulin-loaded CANPs were 72.8 ± 2.1% and 9.9 ± 1.5%, respectively [55]. However, the CANPs were larger than in the present study, measuring 781 ± 61 nm. To achieve successful PH delivery, the CANPs system needed to exhibit sufficient encapsulation efficiency and loading capacity. This ensures the optimal functionality of the entrapped PH at the target site, specifically the deeper layers of the skin, for cosmetic application.

### 3.3. Stability Profiles of CANPs and PH-Loaded CANPs

#### 3.3.1. Physical Stability

**Figure 2:**
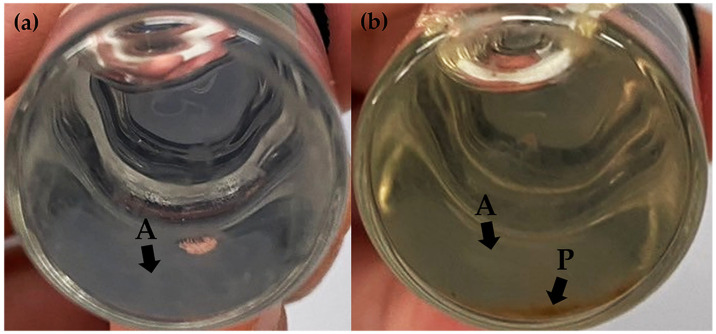
The physical appearance of chitosan alginate nanoparticles (CANPs) formulation F3 (**a**) and *A. domesticus* protein hydrolysate-loaded CANPs formulation F6 (**b**) after a 3-month storage period. The letters A represent the agglomeration of the CANP particles observed as cloudy area at the bottom of the container, and P represents the precipitation of the CANP particles.

The stability profiles of F1, F2, F3, F4, F5, and F6 concerning their external appearance after exposure to heating–cooling cycles and long-term storage conditions were monitored. F1, F2, F4, and F5 exhibited no discernible differences in external appearance after undergoing eight cycles of heating–cooling and long-term storage. This could be attributed to the stabilizing effect of the polyelectrolyte complex formed by sodium alginate and chitosan [39]. In contrast, F3 and F6 exhibited agglomeration and precipitation at the bottom of the bottles after 3 months of storage due to their larger particle size and excess polymer as shown in Figure 2a,b, respectively. The cloudy appearance or even precipitations were detectable with the naked eye, indicating visible signs of instability in both formulations. Similar results were observed in a prior study, where an increase in the relative proportion of sodium alginate or chitosan was found to correlate with an increase in particle size and a subsequent decrease in stability [66]. Specifically, a higher concentration of chitosan resulted in the formation of turbid and cloudy precipitation. Therefore, F3 and F6 were excluded from this study due to their physical instability. 

**Figure 3:**
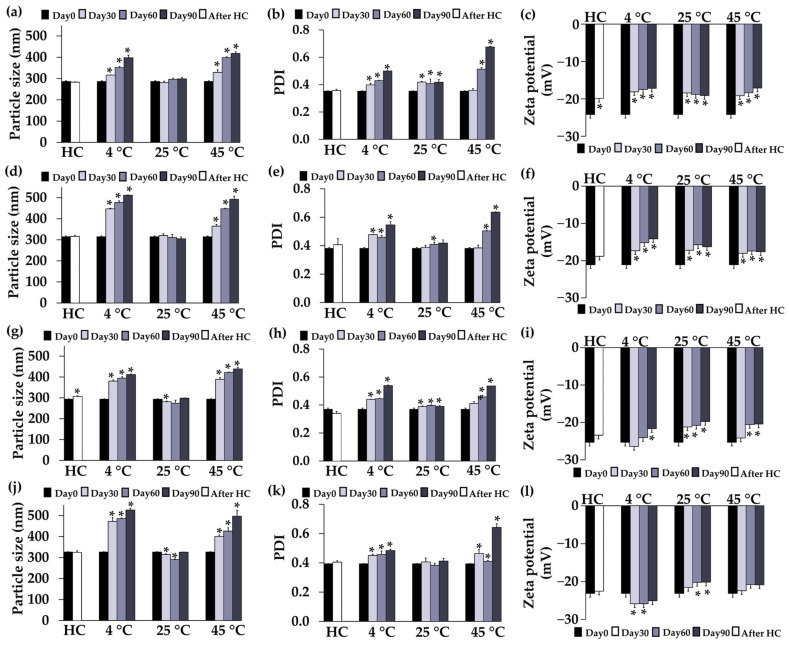
Physical stability profiles, including particle size (**a**), polydispersity index (PDI) (**b**), and zeta potential (**c**) of chitosan alginate nanoparticles (CANPs) formulation F1; particle size (**d**), PDI (**e**), and zeta potential (**f**) of F2; particle size (**g**), PDI (**h**), and zeta potential (**i**) of *A. domesticus* protein hydrolysate-loaded CANPs F4; particle size (**j**), PDI (**k**), and zeta potential (**l**) of F5, before storage (Day 0), after 8 heating–cooling cycles (HC), and after long-term storage at 4 °C, 25 °C, and 45 °C for 1, 2, and 3 months. Asterisks (*) denote significant differences in particle size, PDI, and zeta potential of each formulation between Day 0 and after the stability test analyzed using a pair-sample *t*-test (*p* < 0.05).

The stability profiles of F1, F2, F4, and F5 regarding particle size, PDI, and zeta potential are shown in Figure 3. None of the formulations showed any significant changes in particle size and PDI after eight cycles of heating–cooling (*p* > 0.05), except for the particle size of F4, indicating robust stability of the systems with a narrow distribution. F1 and F2 retained their particle size over 90 days, while F4 and F5 exhibited a slight decrease in particle size on days 30 and 60 under storage conditions at 25 °C. However, at 4 °C and 45 °C conditions, there was a noticeable increase in sizes and PDI starting from the first month and continuing over time. The reason might be due to the temperature-dependent change in polymer solubility [67]. Consequently, it is recommended to store CANPs and PH-loaded CANPs formulations at 25 °C due to their favorable stability. This finding was supported by Abnoos et al. (2018) who demonstrated that the average diameter values of CANPs containing pirfenidone remained consistent over 6 months at room temperature by assessing the particle with a scanning electron microscope [68]. Additionally, the zeta potential of all formulations remained stable after accelerated storage conditions, except for F1, indicating the stability of the colloidal dispersions. Nevertheless, all formulations exhibited a significant increase in zeta potential at all temperatures in long-term storage conditions (*p* < 0.05). This change could be attributed to the protonation of the COO^−^ groups of sodium alginate over time in the acidic environment [69]. The pH condition during CANPs and PH-loaded CANPs preparation closely approached the pKa value of sodium alginate (~3.5). Consequently, the COO^−^ groups underwent protonation, transforming into COOH (unionized forms), leading to an overall progressive increase in zeta potential. 

#### 3.3.2. Chemical Stability

**Figure 4:**
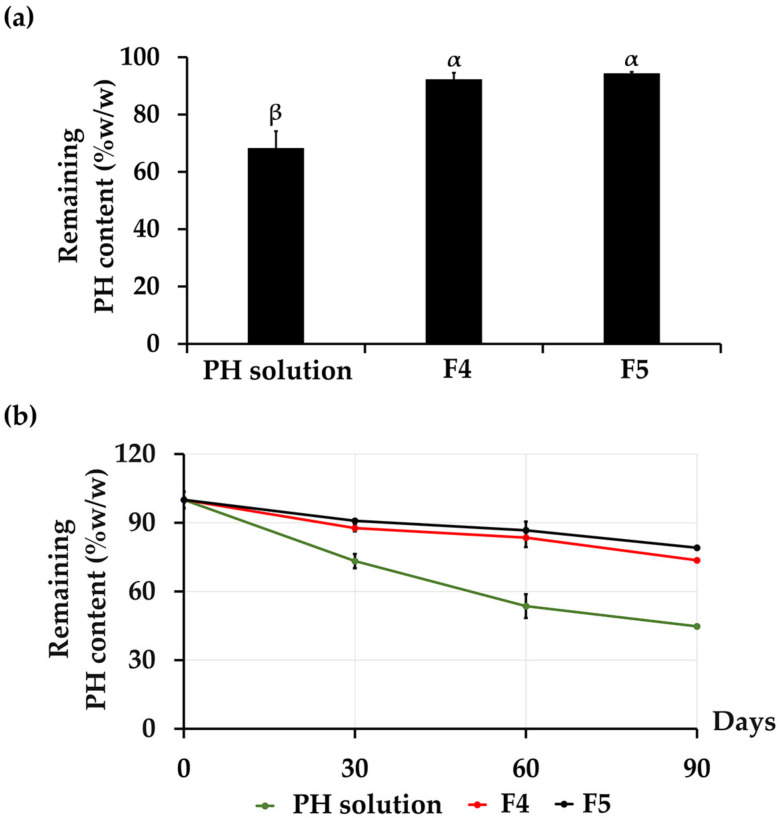
Chemical stability profiles of the various *A. domesticus* protein hydrolysate (PH) formulations, including the remaining protein content of PH after 8 heating–cooling cycles (HC) (**a**) and the remaining protein content of PH after long-term storage at 25 °C for 1, 2, and 3 months from PH aqueous solution (green line), PH-loaded chitosan alginate nanoparticles (CANPs), including formulations F4 (red line) and F5 (black line) (**b**). Different letters (α and β) denote significant differences in the protein content of PH among various PH formulations using a One-way ANOVA followed by a post hoc Tukey test (*p* < 0.05).

The chemical stabilities of PH from aqueous PH solution, F4, and F5 are shown in Figure 4. The remaining total protein content in each formulation was evaluated by the BCA assay due to its capacity to encompass the overall protein content, including a variety of amino acids. This aligns with the precise measurement of protein levels within the PH. The results demonstrated that the incorporation of PH into CANPs significantly enhanced its chemical stability compared to an aqueous PH solution (*p* < 0.05). For an aqueous solution of PH, the protein content deteriorated from the initial state to 68% after undergoing eight cycles of heating–cooling conditions. Nevertheless, the accelerated stability test revealed a notable improvement in the stability of PH with the incorporation into both F4 and F5, with a protein content of 92 ± 2% and 94 ± 1%, respectively, as presented in Figure 4a. Due to the physical instability of CANPs and PH-loaded CANPs at 4 °C and 45 °C in the long-term study, the chemical stability of PH in PH-loaded CANPs was investigated at 25 °C for 1, 2, and 3 months. It was noted that CANPs again augmented the stability of PH. The protein content of F4 was found to be 88 ± 4%, 83 ± 2%, and 74 ± 4%, as well as 91 ± 4%, 87 ± 0%, and 80 ± 4% for F5, while the protein content of PH aqueous solution gradually decreased over time to 73 ± 1%, 54 ± 3%, and 44 ± 5% after 1, 2, and 3 months, respectively, as illustrated in Figure 4b. It was evident that encapsulation effectively stabilized the PH, preventing its degradation. The improved stability could be attributed to the inner location of PH within the polyelectrolyte complex of both polymers, where the polymer matrix of CANPs acted as a protective shell, safeguarding PH from external factors such as heat and oxidative stress [61]. This result aligned with a previous study that reported a notable improvement in the stability of entrapped α-amylase within chitosan–alginate polyelectrolyte complex beads compared to the marketed formulation and the bulk quantity of α-amylase with the shelf-lives of 3.68 years, 0.99 years, and 0.41 years, respectively. [70]. Another study also showed that CANPs protected curcumin diethyl diglutarate from degradation, particularly through oxidation reactions accelerated by heating [71]. Hence, CANPs stand out as a highly promising and effective delivery system, ensuring preservation of PH stability. However, the current study investigated only the total protein content after the stability test. Therefore, in-depth details regarding protein decomposition that might occur during the test are suggested. Further evaluation of the amino acid profiles or investigations using methods that provide detailed insights into the structural integrity and composition of the proteins, such as sodium dodecyl-sulfate polyacrylamide gel electrophoresis (SDS-PAGE), are currently under way to evaluate protein decomposition after the stability test. Furthermore, incorporating FT-IR analysis would offer more in-depth insights into the chemical properties during long-term stability studies. Therefore, it was recommended for further FT-IR investigations of the PH-loaded CANPs.

### 3.4. Characterization of CANPs and PH-Loaded CANPs

#### 3.4.1. External Appearance and Morphology

**Figure 5:**
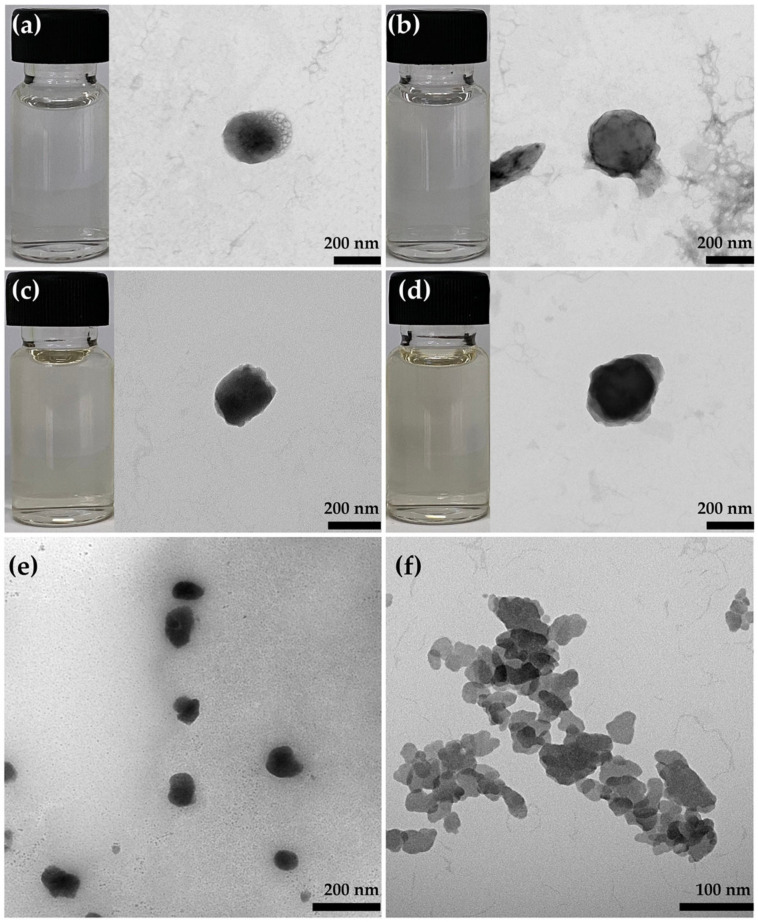
External appearances and TEM micrographs at 25k× magnification of CANPs: formulations F1 (**a**) and F2 (**b**); *A. domesticus* protein hydrolysate-loaded CANPs: formulations F4 (**c**) and F5 (**d**). Overall morphology of F5 at 25k× magnification (**e**) and agglomeration in F5 at 50k× magnification (**f**).

The CANPs (F1 and F2) and PH-loaded CANPs (F4 and F5) formulations that passed the stability assessment were chosen for additional characterization involving the external appearance and morphology under the TEM, as shown in Figure 5. F1 and F2 displayed a uniform and homogeneously translucent liquid appearance, while F4 and F5 consistently exhibited a translucent liquid appearance but with a subtle pale brownish hue, reminiscent of the characteristic color associated with PH. The TEM micrographs of both empty CANPs (F1 and F2) and PH-loaded CANPs (F4 and F5) exhibited distinct and unique appearances, which could be attributed to the rapid solidification that occurs when dropping calcium chloride into the sodium alginate solution [72]. More opacity was observed in the PH-loaded CANPs, suggesting the incorporation of the PH within the CANPs. Additionally, TEM results provided a rough estimation of CANPs’ and PH-loaded CANPs’ size in the nanometer range, corroborating the sizes obtained from the DLS method. A higher concentration of chitosan in the formulation, which resulted in larger particle sizes of CANPs, was confirmed by the TEM micrographs. F2, which contained more chitosan, exhibited larger CANPs compared to F1. Similar results were also observed after the incorporation of PH, as seen in F4 and F5. In addition, a uniform distribution of CANPs is depicted in Figure 5e, while agglomerated particles were also detected, as shown in Figure 5f. The observed agglomeration may be attributed to the intermolecular bonding of polyelectrolyte complexation, forming a network of hydrophobic interactions upon drying in the TEM sample preparation [70]. In conclusion, the preparation method used in this study was found to be effective in producing both CANPs and PH-loaded CANPs.

#### 3.4.2. FT-IR Results

**Figure 6:**
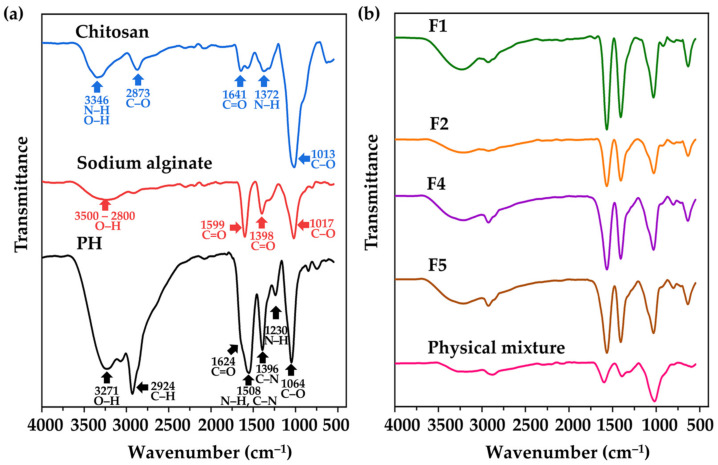
Fourier transform infrared (FT-IR) spectra of pure materials (**a**), including chitosan (blue line), sodium alginate (red line), and *A. domesticus* protein hydrolysate (PH) (black line); chitosan alginate nanoparticles (CANPs) (**b**), including formulations F1 (green line) and F2 (orange line), PH-loaded CANPs, including F4 (purple line) and F5 (brown line), and physical mixture (pink line).

The functional groups, chemical structures, and interactions among substances within CANPs were identified by assessing their infrared light absorption through FT-IR spectroscopy. The FT-IR spectra of starting material (chitosan, sodium alginate, and PH), F1, F2, F4, F5, and its physical mixtures are illustrated in Figure 6. In the chitosan spectra, characteristic peaks at 3346 cm^−1^ were detected indicating N–H stretching vibration overlapping with O–H stretching vibration [39]. Additional peaks were observed at 2873^−1^ (C–O stretch band), 1641 cm^−1^ (C=O stretching of the amide I bond), 1372 cm^−1^ (N–H bend of the amide II bond), and 1013 cm^−1^ (–C–O– stretching vibration linked to the β-d-glucosamine unit of polysaccharides) [73]. For sodium alginate, two distinctive peaks were noted at 1599 cm^−1^ and 1398 cm^−1^ representing asymmetric and symmetric stretching of C=O, respectively [74]. A broad peak at the range of 3500 to 2800 cm^−1^ corresponding to the O–H stretching of the hydroxyl group, and a peak at 1017 cm^−1^ indicating the stretching of C–O groups were also detected [75]. Regarding PH, the active functional group exhibited the peaks observed at 3271 cm^−1^ (O–H stretch), 2924 cm^−1^ (C–H stretch), 1624 cm^−1^ (C=O stretch), 1508 cm^−1^ (C–N stretch), 1508 cm^−1^ (N–H bend), 1396 cm^−1^ (C–N stretch), 1230 cm^−1^ (N–H bend), and 1064 cm^−1^ (C–O stretch) [76,77,78]. 

During CANPs preparation, polyelectrolyte complexation between sodium alginate and chitosan occurred, leading to the disappearance of some characteristic peaks of chitosan and sodium alginate, while new peaks emerged [61]. The FT-IR spectra of F1 and F2 revealed peaks in the range of 3600 to 3000 cm^−1^, corresponding to –OH and –NH_2_ stretching vibrations, as shown in Figure 6b [79]. Some spectral shifts were observed, notably the amide I band shifting from 1641 to 1568 cm^−1^, and the amide II band shifting from 1372 to 1405 cm^−1^. These shifts indicated a successful interaction between the COO^−^ group of sodium alginate and the NH_3_^+^ group of chitosan through electrostatic interaction, as documented by Kumara et al. (2015) [80]. After PH incorporation was observed in CANPs (F4 and F5), similar peak patterns to the empty CANPs spectra (F1 and F2) were observed, implying compatibility of PH with CANPs [81]. This finding is consistent with the observations of Chen et al. (2022), where the spectra of CANPs loaded with clam heparinoid DG1 closely resembled those of empty CANPs [43]. Moreover, in the FT-IR spectra of F4 and F5, the presence of peaks at 2925 cm^−1^ and 2920 cm^−1^, corresponding to the characteristic peaks of PH, strongly indicates the effective entrapment of PH within the CANPs formulation [58]. On the other hand, the physical mixture displayed spectra reminiscent of the pure combination with PH and no observable spectral shifts. This included notable features such as a peak at 1393 cm^−1^ attributed to the amide II bond of PH, a peak at 1597 cm^−1^ corresponding to the C=O of sodium alginate, and a peak at 1019 cm^−1^ indicative of C–O stretching vibration from chitosan. The lack of spectral changes implies that there was no significant interaction in the physical mixture [58]. 

#### 3.4.3. DSC Results

**Figure 7:**
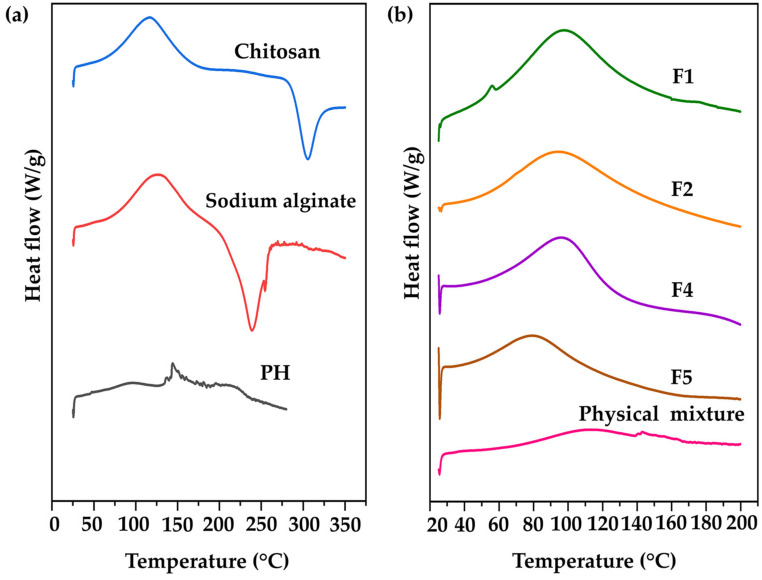
Differential scanning calorimetry (DSC) thermograms of pure material (**a**), including chitosan (CS) (blue line), sodium alginate (SA) (red line), and *A. domesticus* protein hydrolysate (PH) (black line); chitosan alginate nanoparticles (CANPs) (**b**), including formulations F1 (green line) and F2 (orange line), PH-loaded CANPs, including F4 (purple line) and F5 (brown line), and physical mixture (PM) (pink line).

DSC has been widely applied in the investigation of numerous phenomena occurring during the thermal heating of polymers, involving glass transition, melting, crystallization, and curing [82]. DSC is employed to characterize transitions in polymers and composites, and the formation of CANPs could alter thermal behaviors compared to the starting material (pure polymers) [43]. The DSC thermograms of chitosan, sodium alginate, PH, F1, F2, F4, F5, and their physical mixture are presented in Figure 7. Endothermic peaks corresponded to water loss from hydrophilic groups in the polymers, while exothermic peaks were attributed to the degradation of polyelectrolytes, involving dehydration, depolymerization reactions, and potentially partial decarboxylation of protonated carboxylic groups along with oxidation reactions [38]. As a result, chitosan showed an endothermic peak at 117 °C and an exothermic peak at 305 °C. Sodium alginate exhibited an endothermic peak at 126 °C and an exothermic peak at 240 °C. Additionally, PH displayed double endothermic peaks at 95 °C and 145 °C as shown in Figure 7a. In the case of F1 and F2, a single endothermic peak was identified at 97 °C and 94 °C, respectively. The shifting of these peaks in the thermograms indicated the interaction between the sodium alginate and chitosan during CANPs formation [58]. It was noted that the concentration of chitosan did not impact the peak shape but caused a shift in the onset temperature. Furthermore, endothermic peaks of F4 and F5 were observed at 94 °C and 79 °C, respectively. These peaks marginally shifted from the empty CANPs formulations, indicating the successful incorporation of PH in CANPs. In contrast, the thermogram of the physical mixture showed the characteristic peak of an isolated polymer combined with PH, exhibiting an endothermic peak at 112 °C and a tailing pattern similar to the PH thermogram. This finding is supported by a previous study that reported a shifting endothermic peak of CANPs incorporating BSA from the starting material [41]. They also observed that the ratio of chitosan to sodium alginate influenced the onset of endothermic peaks. 

### 3.5. Irritative Potential of CANPs and PH-Loaded CANPs

**Figure 8:**
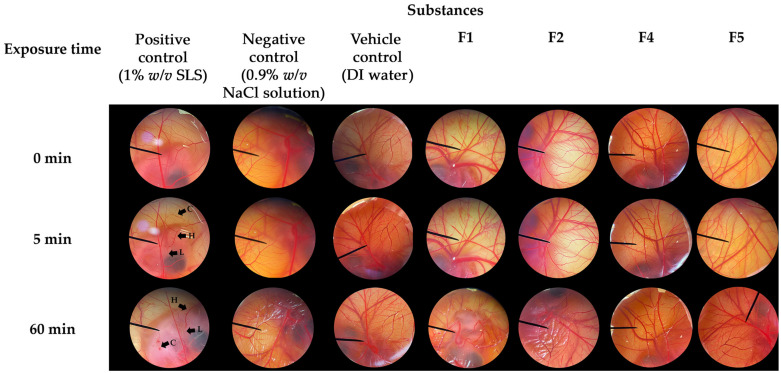
Effect of positive control (1% *w*/*v* sodium lauryl sulfate (SLS) aqueous solution), negative control (0.9% *w/v* sodium chloride (NaCl) solution), vehicle control (DI water), chitosan alginate nanoparticles (CANPs), including formulations F1 and F2, *A. domesticus* protein hydrolysate (PH)-loaded CANPs, including F4 and F5, on the chorioallantoic membrane at 0, 5, and 60 min after exposure.

The irritative potential of F1, F2, F4, and F5 was evaluated using the HET-CAM assay. The results are illustrated in Figure 8 and the irritative levels are categorized as IS presented in Table 3. The validity of the HET-CAM test in the present study has been confirmed. In the assay, an aqueous solution of 1% *w*/*v* SLS, employed as a positive control, induced severe irritation with an IS of 18 ± 1. Vascular lysis, hemorrhage, and coagulation were observed within 5 min after exposure to SLS, as depicted in Figure 8. Consequently, all the irritative signs were pronounced after 60 min of exposure. In contrast, a normal saline solution (0.9% *w*/*v* NaCl) and DI water, used as a negative control and vehicle control, respectively, resulted in no irritation. Remarkably, none of the CANPs or PH-loaded CANPs formulations exhibited any signs of irritation on the CAM. Therefore, F1, F2, F4, and F5 were deemed safe formulations, affirming their suitability for topical applications.

### 3.6. Release Profiles of PH-Loaded CANPs

**Figure 9:**
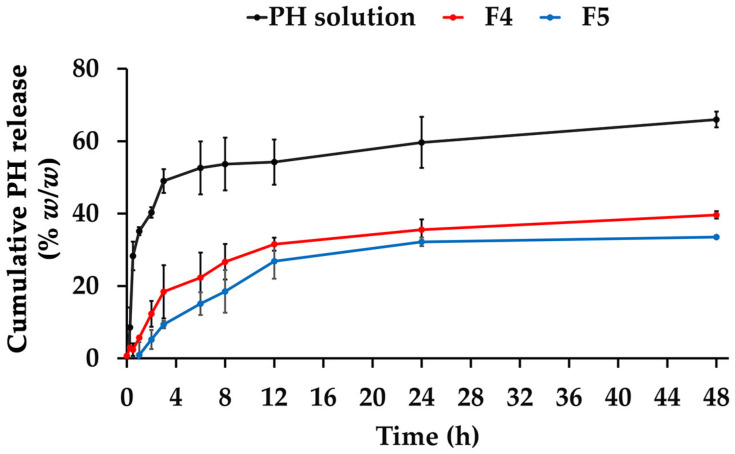
Release profiles of *A. domesticus* protein hydrolysate (PH) over 48 h from PH solution (black line), PH-loaded chitosan alginate nanoparticles (CANPs), including formulations F4 (red line), and F5 (blue line).

The release patterns of the PH solution, F4, and F5 are shown in Figure 9. The initial release pattern was observed in the solution PH, with a rapid release of PH of around 30% within the first hour and up to 50% within 3 h. A significantly different released amount of the PH solution was observed compared to PH-loaded CANPs. Both F4 and F5 displayed similar sustained release patterns with gradual, continuous, and slow release profiles. However, the release rate of F4 and F5 was at its peak during the initial period and gradually decreased over time. This finding supported the assumption that some PH was deposited on the surface of CANPs, resulting in the desorption of proteins from the particle surface and rapid release in the early phases [41]. Moreover, the controlled release patterns were attributed to the polymer matrix layers of CANPs, acting as a hindrance and decelerating the diffusion of encapsulated compounds, resulting in a gradual release over time [63]. Additionally, the significantly highest PH amount released after 48 h study was observed in the PH solution, followed by F4 and F5 (*p* < 0.05), with the cumulative PH release of 66 ± 2%, 40 ± 2%, and 34 ± 0%, respectively. The differences could be attributed to varying chitosan concentrations in producing CANPs, resulting in different porosity and compact structures of CANPs [38]. The release mechanism of entrapped compounds in CANPs could be explained by the diffusion of water into the nanoparticles, swelling of the polysaccharide matrix, and loosening of their compact structure, allowing bioactive molecules to dissociate and diffuse through resulting surface pores [41]. These mechanisms occur simultaneously or in a sequential manner. The results of the present study were consistent with a previous study showing spontaneous release of BSA from CANPs within the first 6 h [41]. Additionally, they also observed that CANPs with lower chitosan/sodium alginate ratio (1:1) displayed a higher release of BSA compared to the other two ratios (2:1 and 1:2), where the release profiles did not reach a constant state.

### 3.7. Skin Permeation and Skin Retention Profiles of PH-Loaded CANPs

**Figure 10:**
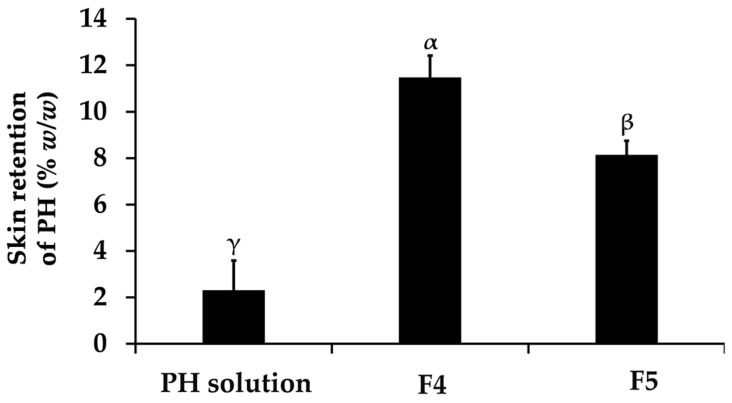
Skin retention profiles of *A. domesticus* protein hydrolysate (PH) from PH solution, PH-loaded chitosan alginate nanoparticles (CANPs), including formulations F4 and F5 after 48 h application. Different letters, α, β, and γ, denote significant differences in the skin retention of PH among the formulations analyzed using a One-way ANOVA followed by a post hoc Tukey test (*p* < 0.05).

The present study revealed that none of the formulations could permeate through the Strat-M^®^ membranes, designed to mimic human skin layers and reach the receptor chamber of the Franz diffusion cell apparatus. Therefore, there were no systemic side effects induced after the topical application of these formulations [45]. However, the skin retention profiles of PH were enhanced by the PH-loaded CANPs formulation, as illustrated in Figure 10. The best formulation, F4, significantly delivered the highest amount of PH into the membrane (12 ± 1%), followed by F5 (8 ± 1%), and PH solution (3 ± 1%), respectively (*p* < 0.05). The limited retention of PH amounts in this study can be ascribed to the hydrophilic nature of both PH and CANPs formulations, which was incongruent with the hydrophobic character of the synthetic membrane designed to simulate the superficial layers of human skin [83]. Consequently, PH became entrapped and accumulated in the inner polymer matrix layer of CANPs rather than diffusing across the synthetic membrane. Nonetheless, CANPs still improved the skin retention of PH compared to the solution. This could be attributed to several properties associated with CANPs, including particle size, enhanced adhesion, controlled release, and protection from external factors [44,84]. These combined mechanisms contributed to the superior skin retention observed with PH-loaded CANPs, especially F4. A previous study also reported that permeation of pirfenidone (PFD) released from CANPs significantly increased compared to PFD solution (*p* < 0.05) [68]. Additionally, fluorescent microscope images of PFD-loaded CANPs labeled with FITC showed that nanoparticles successfully penetrated the skin. Therefore, the CANPs formulation provides a new approach for the dermal delivery of PH, particularly F4, to delivery to target site and ensure effectiveness. Nonetheless, it is recommended that further investigations be conducted in various skin models with epidermal and dermal structures to assess the percutaneous penetration of CANPs into the dermis, focusing particularly on the depth of skin penetration. This will enhance the accuracy of dermal delivery evaluations. Additionally, the implementation of clinical studies is suggested to further validate these findings.

## 4. Conclusions

This study successfully generated CANPs incorporating PH under mild conditions, resulting in favorable characteristics, safety, and robust stability during both accelerated testing and three-month storage. Furthermore, CANPs formulations demonstrated substantial potential in improving the stability and skin delivery of PH, outperforming PH solutions. Additionally, CANPs incorporating PH showed no irritation on the CAM, underscoring their safety for topical use. Despite the initial burst release observed within the initial 3 h, sustained release patterns of PH-loaded CANPs were noticed suggesting their promising application for prolonged and targeted delivery. Moreover, this study highlighted the notable skin retention capabilities of PH in CANPs formulations, particularly F4, compared to PH solution. This research unveils the potential of CANPs formulation as a promising strategy to enhance the skin delivery and stability of protein hydrolysate derived from *A. domesticus* envisioning its application for topical use in the cosmetic or cosmeceutical industry. The findings introduce a novel approach that significantly contributes to the application of PH derived from *A. domesticus* by leveraging advanced nanotechnology to create novel topical formulations, enhancing stability and efficacy through the innovative use of insect-derived PH combined with nanotechnology. Future investigations should explore pharmacokinetics, skin retention through fluorescence microscopy, cytotoxicity, and clinical studies for a comprehensive evaluation of PH-loaded CANPs. Further evaluation of the chemical properties during long-term stability studies of PH-loaded CANPs was recommended, suggesting the utilization of HPLC and FT-IR for in-depth analysis.

## Figures and Tables

**Table 1:** Effects of the concentrations of sodium alginate, calcium chloride, and chitosan on CANPs characteristics.

Sodium Alginate Concentration (mg/mL)	Calcium Chloride Concentration (mg/mL)	Chitosan Concentration (mg/mL)	Size (nm)	PDI	Zeta Potential (mV)
0.1	1.3	0.2	240 ± 7 ^a^	0.61 ± 0.02 ^c^	−16.0 ±0.2 ^c^
0.2	1.3	0.2	258 ± 11 ^b^	0.45 ± 0.01 ^b^	−18.8 ±0.6 ^b^
0.4	1.3	0.2	283 ± 4 ^c^	0.39 ± 0.01 ^a^	−20.0 ±0.1 ^b^
0.6	1.3	0.2	288 ± 5 ^c^	0.40 ± 0.01 ^a^	−27.8 ±2.3 ^a^
0.8	1.3	0.2	319 ± 5 ^d^	0.40 ± 0.01 ^a^	−29.5 ±1.0 ^a^
1.0	1.3	0.2	330 ± 2 ^d^	0.41 ± 0.01 ^a^	−30.2 ± 1.2 ^a^
0.6	1.3	0.2	302 ± 2 ^b^	0.41 ± 0.01 ^b^	−25.3 ± 0.3 ^c^
0.6	1.6	0.2	301 ± 8 ^b^	0.40 ± 0.00 ^b^	−24.7 ± 0.3 ^c,d^
0.6	1.8	0.2	269 ± 5 ^a^	0.29 ± 0.00 ^a^	−27.8 ± 0.1 ^a^
0.6	2.0	0.2	290 ± 3 ^b^	0.42 ± 0.01 ^b^	−26.7 ± 0.1 ^b^
0.6	2.2	0.2	320 ± 8 ^c^	0.41 ± 0.02 ^b^	−25.5 ± 0.4 ^c^
0.6	2.4	0.2	334 ± 3 ^c^	0.45 ± 0.01 ^c^	−24.3 ± 0.6 ^d^
0.6	1.8	0.1	275 ± 5 ^a^	0.35 ± 0.00 ^a^	−24.9 ± 0.8 ^a^
0.6	1.8	0.2	284 ± 4 ^a,b^	0.36 ± 0.01 ^a^	−23.6 ± 0.3 ^a^
0.6	1.8	0.4	295 ± 7 ^b^	0.36 ± 0.01 ^a^	−19.6 ± 0.6 ^b^
0.6	1.8	0.6	330 ± 1 ^c^	0.44 ± 0.00 ^b^	−18.9 ± 0.4 ^b^
0.6	1.8	0.8	356 ± 8 ^d^	0.47 ± 0.01 ^c^	−17.5 ± 0.3 ^c^
0.6	1.8	1.0	393 ± 4 ^e^	0.50 ± 0.01 ^d^	−17.2 ± 0.1 ^c^

NOTE: CANPs = chitosan alginate nanoparticles and PDI = polydispersity index. The data are shown as the mean ± SD (*n* = 3). The small case letters (a, b, c, d and e) indicate significant variability among the CANP formulations developed using different concentrations of sodium alginate, calcium chloride, and chitosan. Statistical significance was analyzed using a One-way ANOVA followed by a post hoc Tukey test (*p* < 0.05).

**Table 2:** Compositions of CANPs and PH-loaded CANPs formulations and their physicochemical characteristics.

Formulation	PH Concentration (mg/mL)	Sodium Alginate Concentration (mg/mL)	Calcium Chloride Concentration (mg/mL)	Chitosan Concentration (mg/mL)	Size (nm)	PDI	Zeta Potential (mV)
F1	-	0.6	1.8	0.1	275 ± 5 ^a^	0.35 ± 0.00 ^a^	−24.9 ± 0.8 ^a^
F2	-	0.6	1.8	0.2	284 ± 4 ^a,b^	0.36 ± 0.01 ^a^	−23.6 ± 0.3 ^a^
F3	-	0.6	1.8	0.4	295 ± 7 ^b^	0.36 ± 0.01 ^a^	−19.6 ± 0.6 ^b^
F4	1.25	0.6	1.8	0.1	309 ± 0 ^c^	0.39 ± 0.01 ^b^	−26.0 ± 0.9 ^a^
F5	1.25	0.6	1.8	0.2	319 ± 1 ^d^	0.40 ± 0.01 ^b^	−24.3 ± 0.1 ^a^
F6	1.25	0.6	1.8	0.4	352 ± 5 ^e^	0.40 ± 0.00 ^b^	−22.6 ± 0.3 ^a^

NOTE: CANPs = chitosan alginate nanoparticles (F1–F3), PH-loaded CANPs = *A. domesticus* protein hydrolysate-loaded chitosan alginate nanoparticles (F4–F6), PH = *A. domesticus* protein hydrolysate, F = formulation, and PDI = polydispersity index. The data are shown as the mean ± SD (*n* = 3). The small case letters (a, b, c, d, and e) indicate significant variability among the formulations. Statistical significance was analyzed using a One-way ANOVA followed by a post hoc Tukey test (*p* < 0.05).

**Table 3:** Irritation scores (IS) and irritation level from HET-CAM assay (*n* =2).

Sample	IS	Irritative Level
Positive control (1% *w*/*v* SLS)	18 ± 1 ^a^	Severe irritation
Negative control (0.9% *w*/*v* NaCl)	0 ± 0 ^b^	No irritation
Vehicle (DI water)	0 ± 0 ^b^	No irritation
F1	0 ± 0 ^b^	No irritation
F2	0 ± 0 ^b^	No irritation
F4	0 ± 0 ^b^	No irritation
F5	0 ± 0 ^b^	No irritation

NOTE: IS = irritation score, SLS = sodium lauryl sulfate, NaCl = sodium chloride, CANPs = chitosan alginate nanoparticles, including formulations F1 and F2, *A. domesticus* protein hydrolysate (PH)-loaded CANPs, including F4 and F5. Different letters (a and b) denote significant differences in the irritation score among various samples using a One-way ANOVA followed by a post hoc Tukey test (*p* < 0.05).

## Data Availability

The original contributions presented in this study are included in the article.
